# Diagnostic yield of esophagogastroduodenoscopy, colonoscopy, and small bowel endoscopy in Thai adults with chronic diarrhea

**DOI:** 10.1186/s12876-021-01998-w

**Published:** 2021-11-06

**Authors:** Julajak Limsrivilai, Choompunuj Sakjirapapong, Onuma Sattayalertyanyong, Tanawat Geeratragool, Phalat Sathirawich, Ananya Pongpaibul, Piyaporn Apisarnthanarak, Phutthaphorn Phaophu, Nichcha Subdee, Phunchai Charatcharoenwitthaya, Nonthalee Pausawasdi

**Affiliations:** 1 Division of Gastroenterology, Department of Medicine, Faculty of Medicine Siriraj Hospital, Mahidol University, 2 Wanglang Road, Bangkoknoi, Bangkok, 10700 Thailand; 2 Department of Pathology, Faculty of Medicine Siriraj Hospital, Mahidol University, Bangkok, Thailand; 3 Department of Radiology, Faculty of Medicine Siriraj Hospital, Mahidol University, Bangkok, Thailand

**Keywords:** Diagnostic yield, Esophagogastroduodenoscopy, Colonoscopy, Small bowel endoscopy, Chronic diarrhea

## Abstract

**Background:**

Gastrointestinal endoscopy is frequently recommended for chronic diarrhea assessment in Western countries, but its benefit in the Southeast Asia region is not well established.

**Methods:**

Medical records of consecutive patients undergoing esophagogastroduodenoscopy (EGD), colonoscopy, and small bowel endoscopy for chronic diarrhea from 2008 to 2018 were reviewed. Small bowel endoscopy included push enteroscopy, balloon-assisted enteroscopy (BAE), and video capsule endoscopy (VCE). The diagnostic yield of each endoscopic modality and predictors for positive small bowel endoscopy were analyzed.

**Results:**

A total of 550 patients were included. The mean age was 54 years, and 266 (46.3%) patients were male. The mean hemoglobin and albumin levels were 11.6 g/dL and 3.6 g/dL, respectively. EGD and colonoscopy were performed in 302 and 547 patients, respectively, and the diagnostic yield was 24/302 (7.9%) for EGD and 219/547 (40.0%) for colonoscopy. EGD did not reveal positive findings in any patients with normal colonoscopy. Fifty-one patients with normal EGD and colonoscopy underwent small bowel endoscopy. Push enteroscopy, BAE, and VCE were performed in 28, 21, and 19 patients with a diagnostic yield of 5/28 (17.9%), 14/21 (66.7%), and 8/19 (42.1%), respectively. Significant weight loss, edema, and hypoalbuminemia were independent predictors for the positive yield of small bowel endoscopy.

**Conclusion:**

Colonoscopy was an essential diagnostic tool in identifying the cause of chronic diarrhea in Thai patients, whereas EGD provided some benefits. Small bowel endoscopy should be performed when colonoscopy and EGD were negative, particularly in patients with significant weight loss, edema, and hypoalbuminemia.

**Supplementary Information:**

The online version contains supplementary material available at 10.1186/s12876-021-01998-w.

## Introduction

Chronic diarrhea affects approximately 5% of the Western population [1]. Functional disorders (e.g., irritable bowel syndrome [IBS]) and inflammatory diseases (e.g., inflammatory bowel disease [IBD], microscopic colitis, and celiac disease) are the most common causes of chronic diarrhea in western countries [2, 3]. In addition to taking a detailed history and performing a complete physical examination, endoscopic evaluation should be considered in patients with inconclusive diagnosis after routine blood and stool tests or who fail to respond to empirical therapy.

According to the American Society for Gastrointestinal Endoscopy (ASGE) guidelines, a diagnostic colonoscopy is recommended for the evaluation of chronic unexplained diarrhea [4]. The diagnostic yield of colonoscopy in patients with chronic diarrhea ranges from 18 to 31% in Western countries, and the common diagnoses are IBD or microscopic colitis [2, 3, 5]. Upper gastrointestinal (GI) evaluation for diseases involving the duodenum should also be considered in chronic diarrhea patients with negative colonoscopy [4]. Celiac disease, giardia infection, Crohn’s disease, eosinophilic gastroenteritis, Whipple’s disease, and intestinal amyloid are probable diagnoses in these patients [4]. Among patients with normal esophagogastroduodenoscopy (EGD) and colonoscopy, video capsule endoscopy (VCE) provides a diagnostic yield ranging from 43 to 54% [6, 7]. Deep enteroscopy with tissue sampling is a potential tool for diagnosing small bowel disease in patients presenting with chronic diarrhea [8–12], but data are limited.

Although gastrointestinal endoscopy is frequently recommended for chronic diarrhea assessment in Western countries, its benefit in the Southeast Asian region is not well established. The etiologies of chronic diarrhea in Southeast Asia differ from those in Western countries. Southeast Asia has a higher prevalence of gastrointestinal infections but a lower prevalence of celiac and IBD [2, 13, 14]. Accordingly, this study aimed to investigate the diagnostic yield of esophagogastroduodenoscopy (EGD), colonoscopy, and small bowel endoscopy in Thai adults with chronic diarrhea.

## Materials and methods

### Study design and population

We retrospectively reviewed the medical records of consecutive patients aged 18 years or older who underwent EGD and/or colonoscopy to investigate chronic diarrhea at Siriraj Hospital, Bangkok, Thailand, from January 2008 to December 2018. We also evaluated consecutive patients with negative EGD and colonoscopy who underwent subsequent small bowel endoscopy, including push enteroscopy (PE), balloon-assisted enteroscopy (BAE), and video capsule endoscopy (VCE) from the same period.

### Ethics approval of research

All methods were carried out in accordance with the Declaration of Helsinki. The protocol for this study was approved by the Siriraj Institutional Review Board (SIRB) on 17 January 2019 (COA No. 045/2019). The requirement to obtain written informed consent from included patients was waived by the Siriraj Institutional Review Board due to the anonymous retrospective nature of this study.

### Data collection

The patients undergoing gastrointestinal endoscopy for chronic diarrhea were identified from our endoscopic unit records. The definition of chronic diarrhea was watery stool > 3 times/day or > 1 occurrence of mucous-bloody stool per day for more than four weeks. Patients who met the criteria were included. We excluded patients with a personal history of underlying intestinal conditions, such as IBD or short bowel syndrome.

### Definition

The positive yield of endoscopy was defined as abnormal findings from either endoscopic or pathologic examination, or both, leading to the diagnosis. The decision to perform tissue biopsies was at the endoscopist’s discretion. Definite diagnosis was made based on standard methods for each particular disease and response to the specific therapy. All patients were required to have at least six months of follow-up duration. The diagnostic yield of each endoscopic modality was the proportion of patients with the positive findings on endoscopy and the total number of patients undergoing that particular endoscopic modality.

### Data analysis and statistical methods

The diagnostic yield of EGD and colonoscopy were analyzed using the whole cohort data. To assess the importance of ileal intubation during colonoscopy, we elected to determine the diagnostic yield of colonoscopy in two methods as follows: (1) including findings from the terminal ileum, and (2) excluding findings from the terminal ileum. The diagnostic yield of small bowel endoscopy was analyzed in patients with negative EGD and colonoscopy. Furthermore, we evaluated the predictive factors for positive small bowel endoscopy in patients with negative EGD and colonoscopy.

Continuous data are presented as mean and standard deviation if normally distributed and as median and range or interquartile range (IQR) if not normally distributed. Categorical variables are presented as frequency and percentage. We assessed the diagnostic yield of each endoscopic modality. Comparison of the diagnostic yield of EGD to colonoscopy and colonoscopy to colonoscopy without ileal intubation was performed using McNemar’s test. Univariate and multivariate analysis to identify predictors of small bowel mucosal diseases in patients with negative EGD and colonoscopy who underwent small bowel endoscopy was performed based on logistic regression. The significant factors in univariate analysis were included in the multivariate analysis. The detail of the variables included in the model and how each variable was coded was available in Additional file 1: Table S3. A two-tailed *p* value < 0.05 was considered statistically significant. All statistical analyses were performed using SAS Statistics software (SAS, Inc., Cary, NC, USA).

## Results

Five hundred and seventy-eight consecutive patients who underwent gastrointestinal endoscopy to investigate chronic diarrhea were identified. Twenty-eight patients with inadequate follow-up time were excluded. A total of 550 patients were included in this analysis. Baseline characteristics are outlined in Table 1. The mean age was 54 years, and 266 (46.3%) patients were male. The mean hemoglobin and albumin levels were 11.63 g/dL (normal 12.0–14.9 g/dL) and 3.59 g/dL (normal 3.5–5.2 g/dL), respectively. The median duration of symptoms at presentation was 12 weeks. The definite diagnoses are shown in Table 2.

**Table 1:** Characteristics of patients in this cohort

Characteristics	(N = 550)
Age (years), mean ± SD	54.16 ± 15.33
Male gender, n (%)	256/550 (46.5%)
Body mass index (kg/m^2^), mean ± SD	21.78 ± 4.84
Comorbidity, n (%)	
Diabetes mellitus	96/550 (17.4%)
Kidney disease	49/550 (8.9%)
Liver disease	46/550 (8.4%)
Human immunodeficiency virus	29/550 (5.3%)
Immunosuppressive agents	58/550 (10.5%)
Presentation, n (%)	
Duration of symptoms (weeks), median (IQR)	12 (5–24)
Diarrhea characters	
Watery	440/550 (80.0%)
Bloody	126/550 (22.9%)
Steatorrhea	10/550 (1.8%)
Abdominal pain	202/550 (36.7%)
Nausea/vomiting	63/550 (10.9%)
Fever	47/550 (8.5%)
Weight loss	307/550 (55.8%)
Significant weight loss (more than 10% of usual weight)	155/550 (28.2%)
Amount of weight loss (kg), median (IQR)	2 (0–7)
Edema	56/550 (10.2%)
Investigations	
Hemoglobin (g/dL), mean ± SD (n = 515)	11.63 ± 2.34
Albumin (g/dL), mean ± SD (n = 431)	3.59 ± 0.99
Stool fat, n (%)	36/110 (32.7%)

*EGD* esophagogastroduodenoscopy, *SD* standard deviation, *IQR* interquartile range

**Table 2:** Etiology of chronic diarrhea in this cohort

Diseases	Number of patients (%) (N = 550)
Infections	
Parasites/Protozoa	27 (4.91%)
Tuberculosis	19 (3.45%)
Cytomegalovirus	19 (3.45%)
* Clostridium difficile* infection*	8 (1.45%)
Bacteria other than *C. difficile*	5 (0.91%)
Neoplasm	
Colonic adenocarcinoma	40 (7.27%)
Gastrointestinal lymphoma	9 (1.64%)
Polyposis syndrome (Peutz-Jeghers syndrome, Cronkhite-Canada syndrome)	2 (0.36%)
Inflammatory bowel disease	
Crohn’s disease	20 (3.64%)
Ulcerative colitis	31 (5.64%)
Microscopic colitis	5 (0.91%)
Systemic autoimmune diseases	
Systemic lupus erythematosus	5 (0.91%)
Behcet’s disease	3 (0.55%)
Drugs	
Nonsteroidal anti-inflammatory agents	14 (2.55%)
Chemotherapy/targeted therapy	9 (1.64%)
Others e.g. colchicine, metformin	7 (1.27%)
Eosinophilic gastroenteritis	24 (4.36%)
Radiation enterocolitis	7 (1.27%)
Ischemic enterocolitis	3 (0.55%)
Graft-versus-host diseases	4 (0.73%)
Gastrointestinal amyloidosis	3 (0.55%)
Small intestinal bacterial overgrowth	21 (3.82%)
Pancreatic diseases	
Chronic pancreatitis	6 (1.09%)
Pancreatic cancer	4 (0.73%)
Status post pancreatic resection	1 (0.18%)
Endocrinologic diseases	
Diabetic diarrhea	16 (2.91%)
Hyperthyroidism	3 (0.55%)
Adrenal insufficiency	2 (0.36%)
Bile salt diarrhea	4 (0.73%)
Lactose intolerance	2 (0.36%)
Irritable bowel syndrome	215 (39.09%)
Idiopathic ulcers of intestine	4 (0.73%)
Idiopathic diarrhea	2 (0.36%)
Others (1 per each etiology): tropical sprue, intestinal lymphangiectasia, systemic mastocytosis, mesenteric neuroendocrine tumor, chronic diverticulitis, portal hypertensive colopathy)	6 (1.09%)

*EGD* esophagogastroduodenoscopy, *SD* standard deviation, *IQR* interquartile range

^*^Presumed *Clostridium difficile* infection was defined as endoscopic findings of pseudomembranous colitis with response to metronidazole or oral vancomycin

### Diagnostic yield of EGD and colonoscopy

**Fig. 1:**
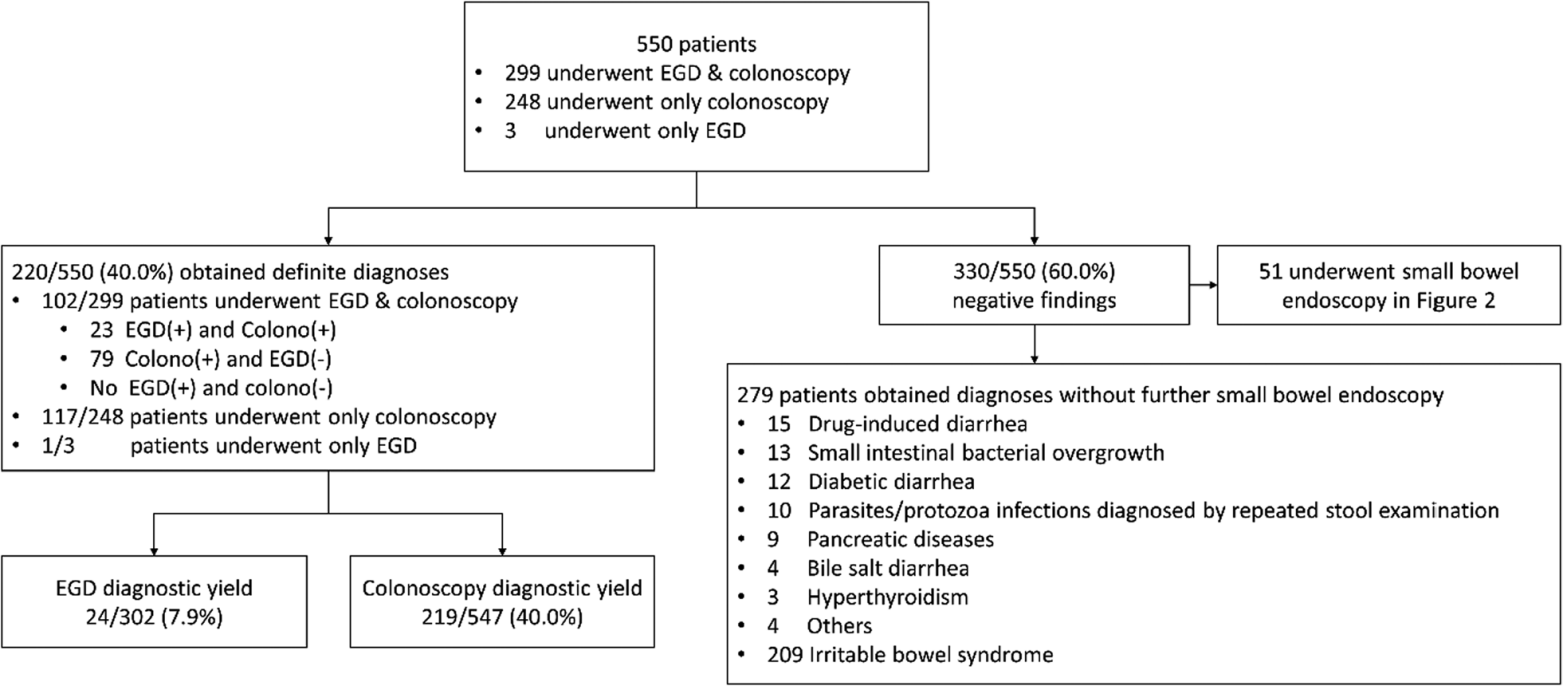
Diagnostic flow diagram in consecutive patients who underwent esophagogastroduodenoscopy and/or colonoscopy. Abbreviation: EGD, esophagogastroduodenoscopy

Of 550 patients, 299 underwent both EGD and colonoscopy, 248 underwent only colonoscopy, and 3 underwent only EGD (Fig. 1). Thus, EGD was performed in 302 patients, and colonoscopy was performed in 547 patients. Among those undergoing EGD, 167/302 (55.3%) had tissue biopsies, and 507/547 (92.7%) patients had tissue biopsies from colonoscopy. A positive yield of endoscopy leading to definite diagnosis was found in 220 of 550 (40.0%) patients. The detail of the definite diagnoses of these 220 patients is available in Additional file 1: Table S1. The common diagnoses included 40/220 (18.2%) colon cancer, 31/220 (14.1%) ulcerative colitis, 18/220 (8.2%) Crohn’s disease, 22/220 (10.0%) eosinophilic colitis, 19/220 (8.6%) ileocolonic tuberculosis, and 18/220 (8.2%) cytomegalovirus ileocolitis. Among the 547 patients who underwent colonoscopy, 219/547 (40.0%) had a positive diagnostic yield. Isolated ileal involvement without colonic lesions was found in 24/547 (4.4%) patients. Therefore, the diagnostic yield would have decreased significantly to 195/547 (35.6%) (*p* < 0.001) had the ileal lesions been excluded. Among the 302 patients who underwent EGD, a positive yield leading to definite diagnosis was found in 24/302 (7.9%) patients, including 5 eosinophilic gastroenteritis, 3 graft-versus-host disease, 3 gastrointestinal lymphomas, 3 cytomegaloviral gastroenteritis, 2 Crohn’s disease, 2 amyloidosis, 2 polyposis syndrome, and one of each following; tuberculosis, NSAID-induced injury, parasitic infestation, and systemic mastocytosis. All patients with positive EGD also had corresponding abnormal colonoscopic findings except for one with lymphoma who underwent only EGD without colonoscopy.

To compare the diagnostic yield of EGD and colonoscopy, subgroup analysis in 299 patients who underwent both EGD and colonoscopy. The diagnostic yield of colonoscopy was 102/299 (34.1%), which is significantly higher than the diagnostic yield of 23/299 (7.7%) of EGD (*p* < 0.001).

### Diagnostic yield of small bowel endoscopy and role of small bowel imaging in patients with negative EGD and colonoscopy

**Fig. 2:**
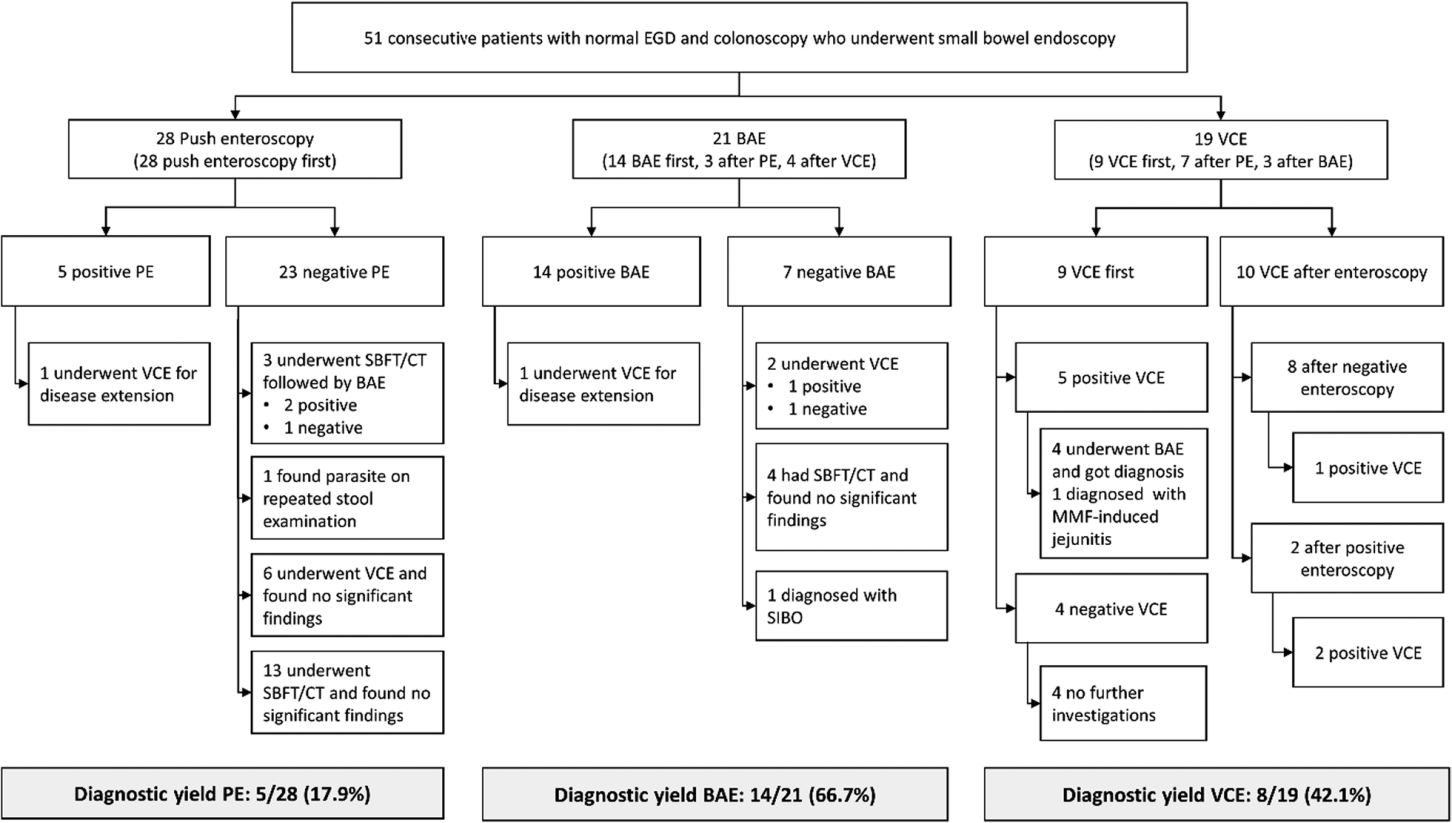
Diagnostic flow diagram of patients with negative esophagogastroduodenoscopy and colonoscopy and underwent subsequent small bowel endoscopy. Abbreviations: BAE, balloon-assisted enteroscopy; CT, computed tomography; EGD, esophagogastroduodenoscopy; MMF, mycophenolate mofetil; PE, push enteroscopy; SBFT, small bowel follow-through; VCE, video capsule endoscopy

Fifty-one consecutive patients with negative EGD and colonoscopy findings undergoing small bowel endoscopy were included. The mean age was 45.8 years. The mean hemoglobin and albumin levels were 10.98 g/dL and 2.75 g/dL, respectively. Push enteroscopy, BAE, and VCE were performed in 28, 20, and 18 patients, respectively. The detail of initial and subsequent diagnostic endoscopic investigations is shown in Fig. 2. Small bowel biopsies were done in all PE and BAE procedures. Twenty-one of 51 (41.2%) patients had positive diagnostic small bowel endoscopy leading to definite diagnoses. The detail of definite diagnoses is shown in Additional file 1: Table S2. The most common diagnosis was parasite and protozoa infection found in 8 (38.10%) patients. The lesions involved the 3^rd^ and 4^th^ part duodenum in 6/21 (28.6%) patients, the jejunum in 21/21 (100%) patients, and the ileum in 5/21 (23.8%) patients. The diagnostic yield of PE, BAE, and VCE was 5/28 (17.9%), 14/21 (66.7%), and 8/19 (42.1%), respectively. For 23 patients with negative PE, 3 had mucosal diseases missed by PE, including 2 intestinal capillariasis and 1 small bowel Crohn's disease. Of these three, two were diagnosed by subsequent BAE, and the other one was diagnosed by repeated stool examination. For 7 patients with negative BAE, only one had a mucosal disease. This patient was diagnosed with a typical VCE finding of intestinal capillariasis and response to anti-parasitic agents. Regarding VCE, as shown in Fig. 2, 9 of 19 patients underwent VCE first; 5 of them had positive findings. Four of 5 patients with positive findings subsequently underwent BAE and obtained a definite diagnose, while the other one was not investigated further and was diagnosed with mycophenolate mofetil-induced jejunitis. Ten of 19 VCE were performed after small bowel enteroscopy; two after positive findings to evaluate disease extension and eight after negative results of small bowel enteroscopy. Among 8 patients with negative small bowel enteroscopy, VCE detected an abnormality in one patient that led to a diagnosis of parasitic infection. Among patients with negative VCE findings, none had mucosal disease.

**Fig. 3:**
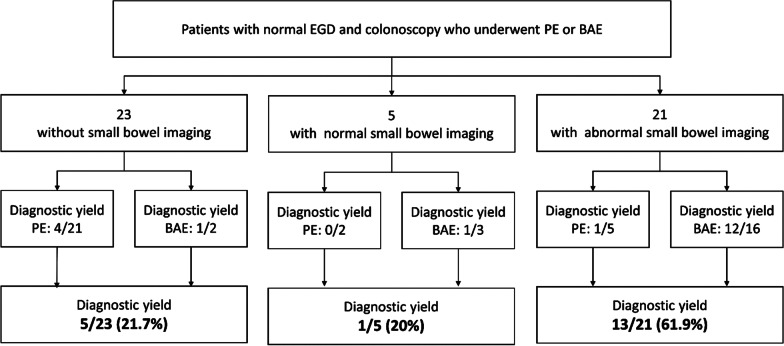
Role of small bowel imaging (small bowel follow-through or computed tomography abdomen) to help localize lesions before small bowel enteroscopy. Abbreviations: PE, push enteroscopy; BAE, balloon-assisted enteroscopy

The role of small bowel imaging to help localize lesions is shown in Fig. 3. Small bowel imaging, either small bowel follow-through (SBFT) or computed tomography (CT) abdomen, was performed before enteroscopy in 7 of 28 push enteroscopy and 19 of 21 balloon-assisted enteroscopy. Among the procedures that had abnormal small bowel imaging performed prior to the procedure, the diagnostic yield was 13/21 (61.9%). The diagnostic yield was 5/23 (21.7%) and 1/5 (20.0%) in the procedures without small bowel imaging and normal small bowel imaging.

### Predictive factors for positive yield of small bowel endoscopy in patients with negative upper and lower endoscopy

Of 330 patients with negative upper and lower endoscopy, 240 patients had complete data of hemoglobin and albumin levels and were included in the analysis. Table 3 shows univariate and multivariate analysis to identify factors that predicted the positive diagnostic findings on small bowel endoscopy. Age younger than 50 years, body mass index lower than 23 kg/m^2^, abdominal pain, significant weight loss of more than 10% of usual body weight, edema, and albumin level lower than 3.5 g/dL were significant factors in univariate analysis, whereas significant weight loss, edema, and low albumin levels remained significant in the multivariate analysis. The low albumin level was the strongest predictor with an odds ratio of 6.68 (95% confident interval 1.59–28.01).

**Table 3:** Univariate and multivariate analysis to identify factors that independently predict positive findings on small bowel endoscopy in patients with negative upper and lower endoscopy

Factors	Univariate analysis	*p* value	Multivariate analysis	*p* value
	Odds ratio (95%CI)		Odds ratio (95% CI)	
Age > 50 years	0.187 (0.07–0.50)	< 0.001	0.37 (0.11–1.22)	0.101
Body mass index < 23 kg/m^2^	10.84 (1.43–82.36)	0.021	6.20 (0.64–60.48)	0.116
Male gender	1.89 (0.76–4.67)	0.167		
Bloody stool	2.23 (0.68–7.24)	0.182		
Steatorrhea	1.52 (0.18–12.99)	0.701		
Abdominal pain	2.68 (1.08–6.66)	0.033	1.34 (0.42–4.29)	0.616
Fever	2.15 (0.24–19.31)	0.494		
Weight loss > 10% of body weight	10.08 (3.52–28.83)	< 0.001	3.80 (1.09–13.20)	0.035
Edema	4.22 (2.10–8.49)	< 0.001	2.71 (1.13–6.50)	0.025
Hemoglobin level < 12 g/dL	1.43 (0.57–3.59)	0.445		
Albumin level < 3.5 g/dL	19.38 (5.49–68.39)	< 0.001	6.68 (1.59–28.01)	0.009

*CI* confidence interval

A *p* value < 0.05 indicates statistical significance

## Discussion

This study found the etiologies of chronic diarrhea in Thai patients different from those in Western patients, particularly in small bowel diseases. The most common cause of small bowel diarrhea in our cohort was parasitic or protozoa infections, while celiac disease, a common cause in western countries [15], was not found. In ileocolonic lesions, although infections such as tuberculosis and cytomegalovirus were still common among Thai patients, the prevalence of IBD was not low, and indeed, it was comparable to the prevalence of chronic infections.

Similar to Western countries, our study showed that colonoscopy had a high diagnostic yield in patients with chronic diarrhea. The diagnostic yield was 40.0%, comparable to the yield reported by several previous studies (range 10.0–49.5%) [2, 16–19]. Furthermore, our study showed that the terminal ileum should be accessed if no lesions were found in the colon. With terminal ileum examination, the diagnostic yield increased from 35.6% to 40.0%. This finding is in accordant with the recommendation from the British Society of Gastroenterology (BSG) [15]. Makkar et al. reported that the diagnostic yield was 15.0% when colonoscopy was performed without ileal intubation, and the yield increased to 16.9% when performed with ileoscopy [20].

ASGE guideline recommends EGD for chronic diarrhea workup when there are no significant laboratory studies and colonoscopy findings [4, 21]. Although the diagnostic yield of EGD is relatively low (7.9%) in this study cohort, it should be offered when colonoscopy is negative as it provides some benefits.

Interestingly, some of the involved small bowel segments, causing chronic diarrhea in our cohort, were at more distal segments unreachable by EGD, most at the jejunum. Twenty-one patients with negative EGD and colonoscopy who underwent small bowel endoscopy were found positive findings leading to a definite diagnosis. The diagnostic yield of push enteroscopy, BAE, and VCE was 17.9% (5/28), 66.7% (14/22), and 42.1% (8/19), respectively. Despite the lowest diagnostic yield, push enteroscopy should be considered when small bowel diarrhea is suspected in our region, given the availability of the test and the less-invasive nature compared to BAE. Balloon-assisted enteroscopy was shown to have a diagnostic yield of 55.0% to 73.5% in previous studies [9–12]. Similarly, the diagnostic yield of BAE in our study was 66.7%. However, BAE should only be performed under the guidance of small bowel imaging or VCE because of its invasiveness. In the present study, the diagnostic yield of VCE was 42.1%, comparable to the reported rate of 42.9–54.5% in previous studies [6, 7]. Furthermore, our study showed that VCE could help to guide the abnormal findings prior to BAE in 4 patients and help to exclude small bowel mucosal lesions if the results were normal since no patients with normal VCE findings were later diagnosed with small bowel mucosal disease.

Our study showed that small bowel imaging studies, either SBFT or CT abdomen, should be considered supplementary investigation to localize the lesion and guide which endoscopic modality should be performed. The diagnostic yield of small bowel enteroscopy appeared to be higher if those procedures were performed in patients with abnormal small bowel imaging (13/21, 61.9%) when compared to the diagnostic yield in patients with no imaging (5/23, 21.7%) and normal imaging (1/5, 20.0%).

For the predictive factors associated with positive small bowel endoscopy among patients with negative EGD and colonoscopy, significant weight loss of more than 10% of usual body weight, albumin levels lower than 3.5 g/dL, and edema were significant predictive factors in multivariate analysis. The hypoalbuminemia was the strongest predictor. This result is in accordant with the study by Song et al., which showed that hypoalbuminemia and hematochezia were significant predictive factors for a positive diagnostic yield of VCE in patients with chronic diarrhea [6].

### Strengths and limitations

The strength of this study was that we reported the diagnostic yield of all endoscopic modalities, including EGD, colonoscopy, and small bowel endoscopy, in patients with chronic diarrhea in Southeast Asia, where the etiologies of chronic diarrhea are different from western countries. We also investigated the role of small bowel imaging in the diagnosis of chronic diarrhea. The most notable limitation was the retrospective design, making it impossible to perform all evaluated modalities in all patients. Tissue biopsies were obtained in 55.3% of patients who underwent EGD, and 92.7% of those underwent colonoscopy, raising concerns of missed diagnoses in some cases. The limitation was minimized by including only patients with adequate follow-up duration to assure that the clinical course supported the definite diagnoses. In this study, the prevalence of IBS was relatively high (39%). IBS could have been over-diagnosed in our study due to the retrospective design and the diagnoses were made based on history documented in medical records. Some physicians noted that patients had chronic diarrhea without the detail of whether their diarrhea was intermittent or persistent. We included all patients with chronic diarrhea longer than 4 weeks based on their medical records. The lack of detailed information of the diarrhea pattern (intermittent vs. persistent) might have resulted in high numbers of patients with IBS. Also, some physicians might diagnose chronic diarrhea without organic diseases with IBS without differentiating IBS from functional diarrhea according to ROME IV criteria [22]. Another limitation was that our data were collected from a single tertiary referral center. The etiology of chronic diarrhea may be different in other general hospitals. Lastly, the sample size was relatively small and may not be representative of the general population.

## Conclusion

Colonoscopy was an essential diagnostic tool in identifying the cause of chronic diarrhea in Thai patients, whereas EGD provided some benefits. Patients with negative colonoscopy and EGD who had clinical features suggestive of small bowel lesions, including significant weight loss, edema, and hypoalbuminemia, required further small bowel evaluation employing small bowel endoscopy.

## Supplementary Information

**Additional file 1. Table S1**. Definite diagnoses made by upper and lower endoscopy.** Table S2**. Definite diagnoses made by small bowel endoscopy.** Table S3**. Code for logistic regression analysis.

## Data Availability

The datasets generated and/or analyzed during the current study are not publicly available due to our center's patient confidentiality policies, but they may be made available by the corresponding author to appropriate parties upon reasonable request.

## Abbreviations

BAE: Balloon-assisted enteroscopy
BMI: Body mass index
CI: Confidence interval
CMV: Cytomegalovirus
COA: Certificate of approval
CT: Computed tomography
DM: Diabetes mellitus
EGD: Esophagogastroduodenoscopy
GI: Gastrointestinal
Hb: Hemoglobin
HIV: Human immunodeficiency virus
IBD: Inflammatory bowel disease
IBS: Irritable bowel syndrome
IQR: Interquartile range
NSAID: Non-steroidal anti-inflammatory drug
SBFT: Small bowel follow through
SLE: Systemic lupus erythematosus
VCE: Video capsule endoscopy
