# Supplementary material for: Diagnostic yield of esophagogastroduodenoscopy, colonoscopy, and small bowel endoscopy in Thai adults with chronic diarrhea
Source: BMC Gastroenterol. 2021 Nov 6;21:417. doi: 10.1186/s12876-021-01998-w (PMC8571819; doi:10.1186/s12876-021-01998-w)
Supplement: Supplementary file 1 — Additional file 1. Table S1. Definite diagnoses made by upper and lower endoscopy. Table S2. Definite diagnoses made by small bowel endoscopy. Table S3. Code for logistic regression analysis. [file 12876_2021_1998_MOESM1_ESM.docx]

**Additional file 1**

Table S1 Definite diagnoses made by upper and lower endoscopy

| **Diseases** | **Number of patients (%)**  **(N=220)** |
| --- | --- |
| Infections |  |
| - Parasites/Protozoa | 8 (3.64%) |
| - Cytomegalovirus | 18 (8.18%) |
| - Tuberculosis | 19 (8.64%) |
| - *Clostridium difficile* infection* | 8 (3.64%) |
| - Bacteria other than *C. difficile* | 5 (2.27%) |
| Neoplasm |  |
| - Colonic adenocarcinoma | 40 (18.18%) |
| - Gastrointestinal lymphoma | 7 (3.18%) |
| - Polyposis syndrome (Peutz-Jeghers syndrome, Cronkhite-Canada syndrome) | 2 (0.91%) |
| Inflammatory bowel disease |  |
| - Crohn’s disease | 18 (8.18%) |
| - Ulcerative colitis | 31 (14.09%) |
| Microscopic colitis | 5 (2.27%) |
| Behcet’s disease | 3 (1.36%) |
| Drugs |  |
| - Nonsteroidal anti-inflammatory agents | 9 (4.09%) |
| - Other drugs | 1 (0.45%) |
| Eosinophilic gastroenteritis | 22 (10.00%) |
| Radiation enterocolitis | 7 (3.18%) |
| Ischemic enterocolitis | 3 (1.36%) |
| Intestinal lymphangiectasia | 1 (0.45%) |
| Graft-versus-host diseases | 4 (1.82%) |
| Gastrointestinal amyloidosis | 3 (1.36%) |
| Systemic mastocytosis | 1 (0.49%) |
| Others | 6 (2.72%) |

Table S2 Definite diagnoses made by small bowel endoscopy

| **Diseases** | **Number of patients (%)**  **(N=21)** |
| --- | --- |
| Parasites/Protozoa | 8 (38.10%) |
| Cytomegalovirus | 1 (4.76%) |
| Gastrointestinal lymphoma | 1 (4.76%) |
| Crohn’s disease | 2 (9.52%) |
| Systemic lupus erythematosus with mucosal vasculitis | 2 (9.52%) |
| Eosinophilic gastroenteritis | 2 (9.52%) |
| Nonsteroidal anti-inflammatory agents | 1 (4.76%) |
| Mycophenolate mycophenolate mofetil | 1 (4.76% |
| Tropical sprue | 1 (4.76%) |
| Bowel congestion from mesenteric neuroendocrine tumor | 1 (4.76%) |
| Idiopathic jejunitis | 1 (4.76%) |

Table S3 Code for logistic regression analysis

| **Factors** |  | **Univariate analysis** | **Multivariate analysis** |
| --- | --- | --- | --- |
| Age > 50 years | Categorical | Yes | Yes |
| Body mass index < 23 kg/m^2^ | Categorical | Yes | Yes |
| Male gender | Categorical | Yes |  |
| Bloody stool | Categorical | Yes |  |
| Steatorrhea | Categorical | Yes |  |
| Abdominal pain | Categorical | Yes | Yes |
| Fever | Categorical | Yes |  |
| Weight loss > 10% of body weight | Categorical | Yes | Yes |
| Edema | Categorical | Yes | Yes |
| Hemoglobin level < 12 g/dL | Categorical | Yes |  |
| Albumin level < 3.5 g/dL | Categorical | Yes | Yes |
